# Identification and validation of immune-related biomarkers and potential regulators and therapeutic targets for diabetic kidney disease

**DOI:** 10.1186/s12920-023-01519-6

**Published:** 2023-05-01

**Authors:** Shengnan Chen, Bo Li, Lei Chen, Hongli Jiang

**Affiliations:** 1 Department of Blood Purification, Kidney Hospital, The First Affiliated Hospital of Xi’an Jiaotong University, West Yanta Road No. 277, Xi’an, 710061 Shannxi China; 2 Department of Nephrology, Ningxia Medical University Affiliated People’s Hospital of Autonomous Region of Ningxia, Yinchuan, 750002 Ningxia China

**Keywords:** Diabetic kidney disease, Immune-related biomarkers, Experimental validation, Transcription factors, microRNAs, Therapeutic drugs

## Abstract

**Background:**

Diabetic kidney disease (DKD) is a major complication of diabetes and the leading cause of end-stage renal disease worldwide. Renal inflammation and infiltration of immune cells contribute to the development and progression of DKD. Thus, the aim of the present study was to identify and validate immune-related biomarkers and analyze potential regulators including transcription factors (TFs), microRNAs (miRNAs), and drugs for DKD.

**Methods:**

Immune-related genes from the ImmPort database and glomeruli samples from GSE1009 and GSE30528 were used to identify differentially expressed immune-related genes (DEIRGs) of DKD. The expression level and clinical correlation analyses of DEIRGs were verified in the Nephroseq database. Murine podocytes were cultured to construct the high glucose-induced podocyte injury model. The reliability of the bioinformatics analysis was experimentally validated by RT-qPCR in podocytes. Networks among DEIRGs, regulators, and drugs were constructed to predict potential regulatory mechanisms for DKD.

**Results:**

DKD-associated DEIRGs were identified. *CCL19* and *IL7R* were significantly upregulated in the DKD group and negatively correlated with glomerular filtration rate (GFR). *GHR*, *FGF1*, *FYN*, *VEGFA*, *F2R*, *TGFBR3*, *PTGDS*, *FGF9*, and *SEMA5A* were significantly decreased in the DKD group and positively correlated with GFR. RT-qPCR showed that the relative mRNA expression levels of *GHR*, *FGF1*, *FYN*, *TGFBR3*, *PTGDS*, *FGF9*, and *SEMA5A* were significantly down-regulated in the high glucose-induced podocyte injury group. The enriched regulators for DEIRGs included 110 miRNAs and 8 TFs. The abnormal expression of DEIRGs could be regulated by 16 established drugs.

**Conclusions:**

This study identified immune-related biomarkers, regulators, and drugs of DKD. The findings of the present study provide novel insights into immune-related diagnosis and treatment of DKD.

**Supplementary Information:**

The online version contains supplementary material available at 10.1186/s12920-023-01519-6.

## Introduction

Diabetic kidney disease (DKD) is a major complication of diabetes and the leading cause of end-stage renal disease worldwide [1] which places an enormous burden on healthcare systems. An increasing number of studies suggest that renal inflammation and infiltration of immune cells contribute to the development and progression of DKD [2]. Therefore, immune-related genes (IRGs) play critical roles in the activation and recruitment of inflammatory and immune cells. Considering the existing treatments for DKD are not fully satisfactory, more investigations on the novel biomarkers, mechanisms, and therapeutic targets are needed to slow the progression of DKD. Targeting immune-related biomarkers may be a promising novel therapeutic approach for DKD. Due to this, identifying IRGs and elucidating their associated regulators is helpful to develop new therapeutic targets for DKD. Transcription factors (TFs) and microRNAs (miRNAs) are prominent regulators of gene expression [3]. In addition, drugs can bind to target genes and influence the biological process and expression of target genes [4]. Based on these theoretical foundations, if a TF, a miRNA, or a drug oppositely modulated the expression of IRGs involved in DKD, that TF, miRNA, or drug may be used for the treatment of DKD.

Thereby, in the current study, we aimed to identify and validate immune-related biomarkers for DKD. Glomerular podocytes are the main component in maintaining the glomerular normal filtration barrier. Therefore, podocytes are the initial target of cellular damage in the incidence of DKD [5]. And the injury and loss of podocytes are important features of DKD [6]. Therefore, we established the high glucose-induced podocyte injury model and performed reverse transcription-quantitative polymerase chain reaction (RT-qPCR) to further verify the credibility of our in silico findings. And the potential regulators, including TFs, miRNAs, and drugs associated with these experimentally validated biomarkers were explored. The findings of the present study may provide potential novel insights into immune-related diagnosis and treatment of DKD.

## Materials and methods

### Data source

The Gene Expression Omnibus (GEO) database is an international public repository of high-throughput gene expression datasets [7]. DKD-related glomerular gene expression profiles (GSE1009 and GSE30528) were downloaded from the GEO DataSets (https://www.ncbi.nlm.nih.gov/geo/). The platform annotation file was used to convert the probe expression matrix into a gene expression matrix. The microarray data of GSE1009 was based on the GPL8300 annotation platform, and GSE30528 was based on the GPL571 platform. The Affymetrix data of GSE1009 contained glomerular samples from 3 healthy controls and 3 DKD patients. GSE30528 dataset encompassed glomerular samples from 9 DKD patients and 13 healthy controls.

ImmPort is an open repository of human immunology database [8]. A list of IRGs was downloaded from the ImmPort database (https://www.immport.org/home).

### Screening for differentially expressed immune-related genes (DEIRGs)

The Bioconductor R package “limma” [9] was used to obtain differentially expressed genes (DEGs) between DKD and normal controls in GSE1009 and GSE30528 datasets. Genes with |Log fold change (FC)| ≥ 1 and adjusted *P* value < 0.05 were defined as DEGs. Then the intersection of the DEGs and the IRGs was selected as DEIRGs. The Venn diagram was created with an online tool jvenn [10].

### The clinical significance of DEIRGs

The clinical significance of these DEIRGs in DKD patients was validated via the Woroniecka Diabetes Glom dataset in Nephroseq v5 online database (http://v5.nephroseq.org). The comparison of DEIRGs expression levels between DKD and healthy control group was performed by unpaired t-test (data with normal distribution and equal variance) and Mann–Whitney U test for non-normally distributed data. The relationship between DEIRGs and glomerular filtration rate (GFR) was analyzed by Pearson’s correlation analysis. *P* < 0.05 (two-tailed) was considered as statistically significant.

### Cell culture

Mouse Podocyte Clone-5 (MPC5) cell lines were purchased from the American type culture collection cell bank. MPC5 cell lines were routinely cultured in Dulbecco’s modified Eagle’s medium (DMEM) containing 10% fetal bovine serum, 100 U/ml penicillin, 100 μg/ml streptomycin, and 0.25 μg/ml amphotericin B and maintained at 37 °C incubator containing 5% CO_2_. The culture medium was changed every 2 days. When 80% confluence was reached, the podocytes were divided into the normal control group (NC group, 30 mmol/l mannitol as an osmotic control) and the high-glucose group (HG group, 30 mmol/l glucose). Mannitol and glucose powder were purchased from Solarbio Technology Co.Ltd and dissolved in DMEM for preparing the working stock concentration of 30 mmol/l. The cells were harvested 72 h after treatment.

### RT-qPCR

Total RNA was extracted from the cells in NC and HG groups by using SteadyPure Universal RNA Extraction Kit II (Accurate Biotechnology Co. Ltd.) according to the manufacturer’s instructions. The complementary DNA was synthesized using Evo M-MLV RT Premix for qPCR AG (Accurate Biotechnology Co. Ltd.). The reverse transcription was performed on a Bio-Rad S1000^TM^ Thermal Cycler (Bio-Rad Laboratories). RT-qPCR was performed on the Bio-Rad CFX-96 Real-time PCR system (Bio-Rad Laboratories) using the SYBR^®^ Green Premix Pro Taq HS qPCR Kit (Accurate Biotechnology Co. Ltd.). The standard amplification parameters for PCR consisted of an initial denaturation step (95 °C for 30 s), followed by 40 cycles of 95 °C for 5 s and annealing at 60 °C for 30 s. All primers for PCR amplification were purchased from Shanghai Sangon Biotechnology and the primer sequences are shown in Table 1. mRNA expression was normalized to beta-actin expression. Then, the relative expression levels of genes were calculated using the 2^−ΔΔCT^ method. All samples were performed in triplicate and the experimental data were presented as mean ± standard deviation. Between-group comparisons were analyzed by unpaired t test. *P* < 0.05 (two-tailed) was considered as statistically significant. DEIRGs validated by RT-qPCR were defined as experimentally validated DEIRGs.

**Table 1:** Primer sequences for RT-qPCR

Gene symbol	Accession number	Forward primer (5′–3′)	Reverse primer (5′–3′)
Beta-actin	NM_007393	TATGCTCTCCCTCACGCCATCC	GTCACGCACGATTTCCCTCTCAG
*CCL19*	NM_011888	GGGTGCTAATGATGCGGAAGACTG	AGCGGAAGGCTTTCACGATGTTC
*IL7R*	NM_008372	AGGAGTTGGAGACACAGGGACAC	CTAACTGTTTCTGGTGGGCTGACTG
*GHR*	NM_010284	ACTTGCCTTATGATGCTTCCCTTGG	AGTTGGTGGGTTGCCTCAGTTTC
*FGF1*	NM_010197	CACAACCTTCGCAGCCCTGAC	TGGTCGCTCCTGTCCCTTGTC
*FYN*	NM_001122893	GACCTCCATCCCGAACTACAACAAC	GTGTCACTCCTGTCCCTCCTCTC
*VEGFA*	NM_001025250	CTTCGCTTACTCTCACCTGCTTCTG	GCTGTCATGGGCTGCTTCTTCC
*F2R*	NM_010169	TGCCTACCTCCTCTGTGTCTGTG	ACTGCTGGGATCGGAACTTTCTTTG
*TGFBR3*	NM_011578	AAAGCCGCCGAAGGTTGTGTC	CTGGAAGGTGCTGTAAGGATTGGAG
*PTGDS*	NM_008963	GGGCTCCTGGACACTACACCTAC	CAGAGCGTACTCGTCATAGTTGGC
*FGF9*	NM_013518	ACTATCCAGGGAACCAGGAAAGACC	ATGCCGAGGTAGAGTCCACTGTC
*SEMA5A*	NM_009154	GGCAAGATCCAGCAGCGTAGAAG	GGCAGTAGGTGTAGACAAGCAGTG

### Construction of the experimentally validated target DEIRGs-miRNAs-TFs network

miRWalk database is an online platform which can be used to predict the interaction between miRNA and mRNA [11]. TargetScan is an experimentally validated miRNA-mRNA interaction database [12]. miRDB is an online database for the prediction of miRNA targets [13]. The validated DEIRGs were uploaded to miRWalk database. To increase the reliability of predictions, the intersection portion of the TargetScan and miRDB database was selected as predicted miRNAs. NetworkAnalyst [14] is a platform that integrates resources from ChEA [15] and JASPAR [16] databases to make predictions about TFs. TFs simultaneously enriched in ChEA and JASPAR databases were recorded. The interaction network between target genes, miRNAs, and TFs was constructed and visualized by Cytoscape software.

### Identification of the drugs target to the experimentally validated DEIRGs

The Comparative Toxicogenomics Database (CTD) [17] is an open online database that can effectively predict the correlation between diseases, drugs, and genes. “Diabetic nephropathies” and “Diabetic kidney diseases” were entered into the CTD database to obtain Chemical-Gene Interactions. Drugs or chemicals that can influence the expression of the experimentally validated DEIRGs were selected to construct the drug-gene interaction network by using Cytoscape software.

## Results

### Identification of DEIRGs

**Fig. 1:**
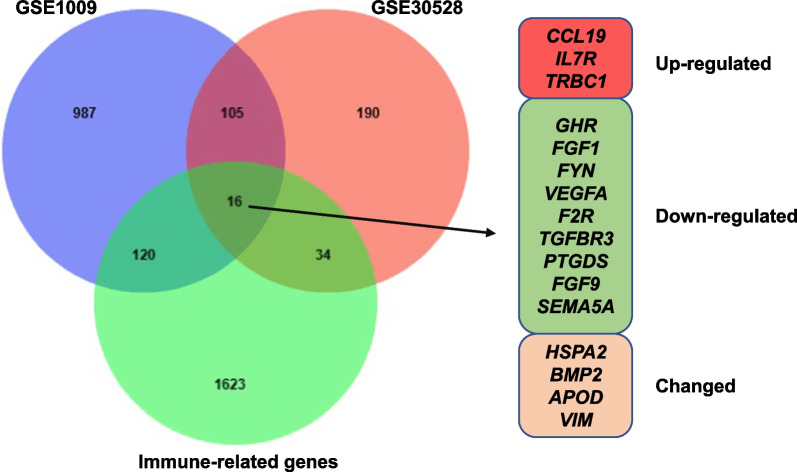
DEIRGs associated with DKD. The blue circle represents the differentially expressed genes (DEGs) of GSE1009, the red circle represents the DEGs of GSE30528, and the green circle represents the immune-related genes. The red box indicates that genes are upregulated in diabetic kidney disease (DKD) group while green box represents downregulated differentially expressed immune-related genes (DEIRGs) in DKD. “Changed” represents the expression of genes changed in opposite direction between GSE1009 and GSE30528

According to the defined criteria, 1228 and 345 DEGs were extracted from GSE1009 and GSE30528 respectively. Subsequently, the intersection of DEGs and IRGs was performed by the Venn diagram to obtain DEIRGs. Altogether, 16 DKD-associated DEIRGs were identified including 3 common upregulated DEIRGs (*CCL19*, *IL7R*, and *TRBC1*), 9 common downregulated DEIRGs (*GHR*, *FGF1*, *FYN*, *VEGFA*, *F2R*, *TGFBR3*, *PTGDS*, *FGF9*, and *SEMA5A*), and 4 changed genes (Fig. 1). The detailed comparison of upregulated and downregulated DEIRGs in GSE1009 and GSE30528 can be seen in Additional file 1: Fig. S1. The common upregulated and downregulated DEIRGs were selected as key DEIRGs for further analysis.

### Validation of the clinical significance of DEIRGs

**Fig. 2:**
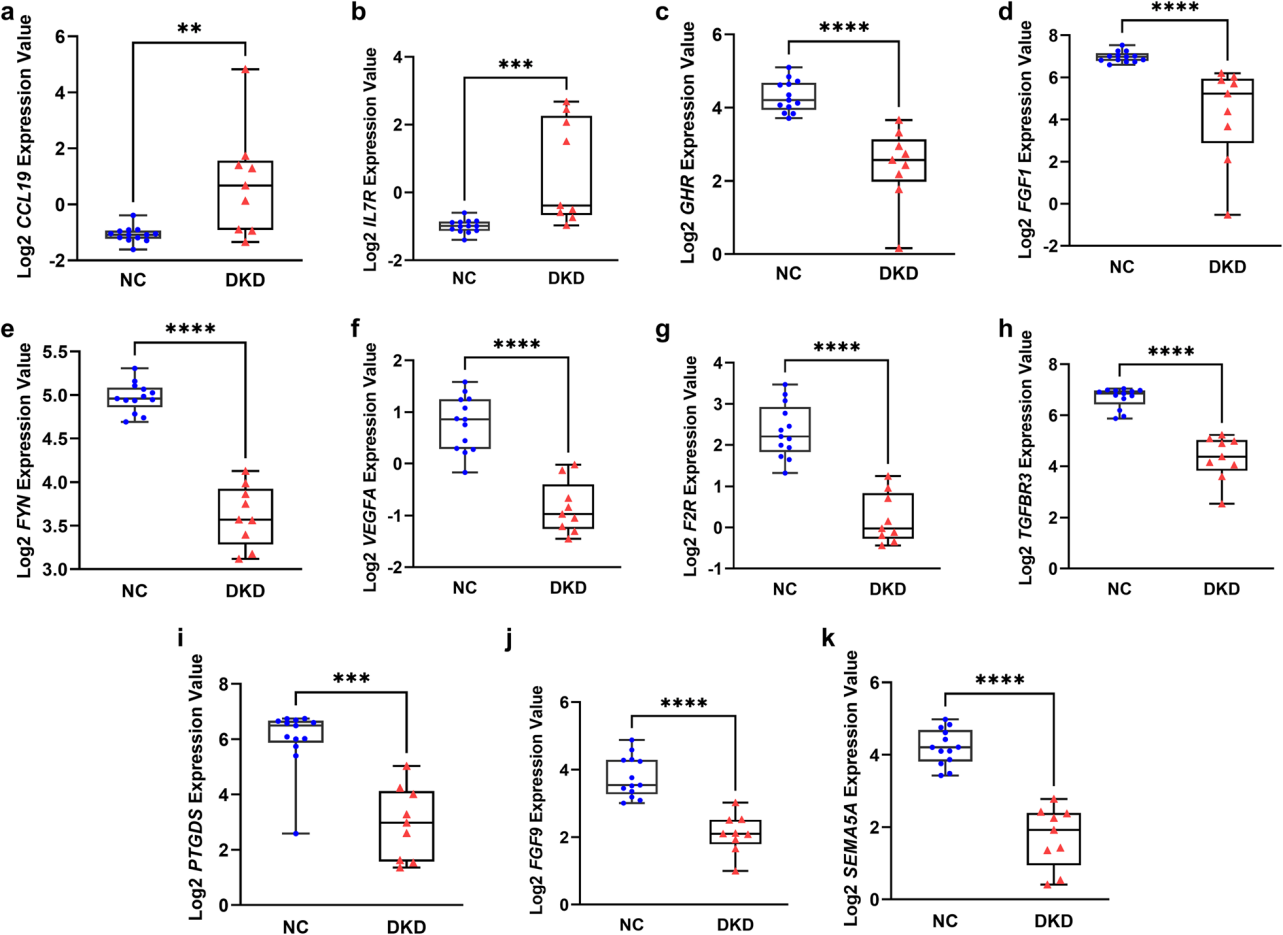
The expression levels of DEIRGs **a** *CCL 19*, **b** *IL7R*, **c** *GHR*, **d** *FGF1*, **e** *FYN*, **f** *VEGFA*, **g** *F2R*, **h** *TGFBR3*, **i** *PTGDS*, **j** *FGF9*, and **k** *SEMA5A* in DKD and NC glomerular tissues. DEIRGs, differentially expressed immune-related genes; NC, normal control; DKD, diabetic kidney disease, ****P < 0.0001; ***P < 0.001; **P < 0.01

The expression profiles of TRBC1 could not be retrieved in the Nephroseq database. *CCL19*, *IL7R*, *GHR*, *FGF1*, *FYN*, *VEGFA*, *F2R*, *TGFBR3*, *PTGDS*, *FGF9*, and *SEMA5A* could be analyzed in the Nephroseq database. The expression levels of *CCL19* and *IL7R* were significantly upregulated in the glomerular tissues of DKD patients compared with healthy living donors (Fig. 2a, b). The expression levels of *GHR*, *FGF1*, *FYN*, *VEGFA*, *F2R*, *TGFBR3*, *PTGDS*, *FGF9*, and *SEMA5A* were significantly downregulated in the DKD glomerular tissues compared with healthy kidney samples (Fig. 2c–k).

**Fig. 3:**
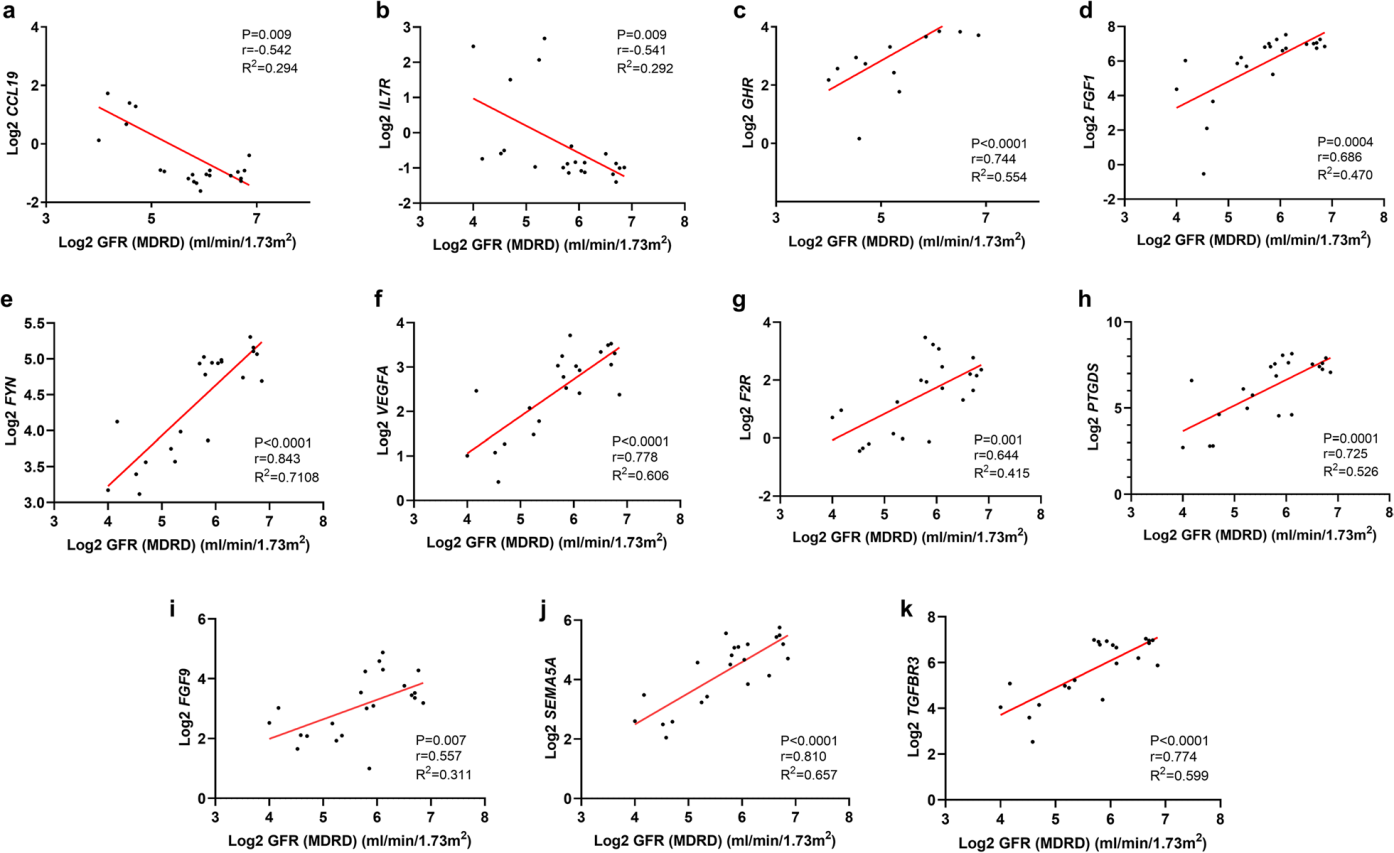
The correlation analysis between DEIRGs **a** *CCL 19*, **b** *IL7R*, **c** *GHR*, **d** *FGF1*, **e** *FYN*, **f** *VEGFA*, **g** *F2R*, **h** *TGFBR3*, **i** *PTGDS*, **j** *FGF9*, **k** *SEMA5A* and GFR. DEIRGs, differentially expressed immune-related genes; *GFR* glomerular filtration rate

The results of correlation analysis showed that *CCL19* and *IL7R* were negatively correlated with GFR (Fig. 3a, b) while *GHR*, *FGF1*, *FYN*, *VEGFA*, *F2R*, *TGFBR3*, *PTGDS*, *FGF9*, and *SEMA5A* were positively correlated with GFR (Fig. 3c–k). Ultimately, the clinical significance of *CCL19*, *IL7R*, *GHR*, *FGF1*, *FYN*, *VEGFA*, *F2R*, *TGFBR3*, *PTGDS*, *FGF9*, and *SEMA5A* were validated.

### RT-qPCR validation of differential gene expression

**Fig. 4:**
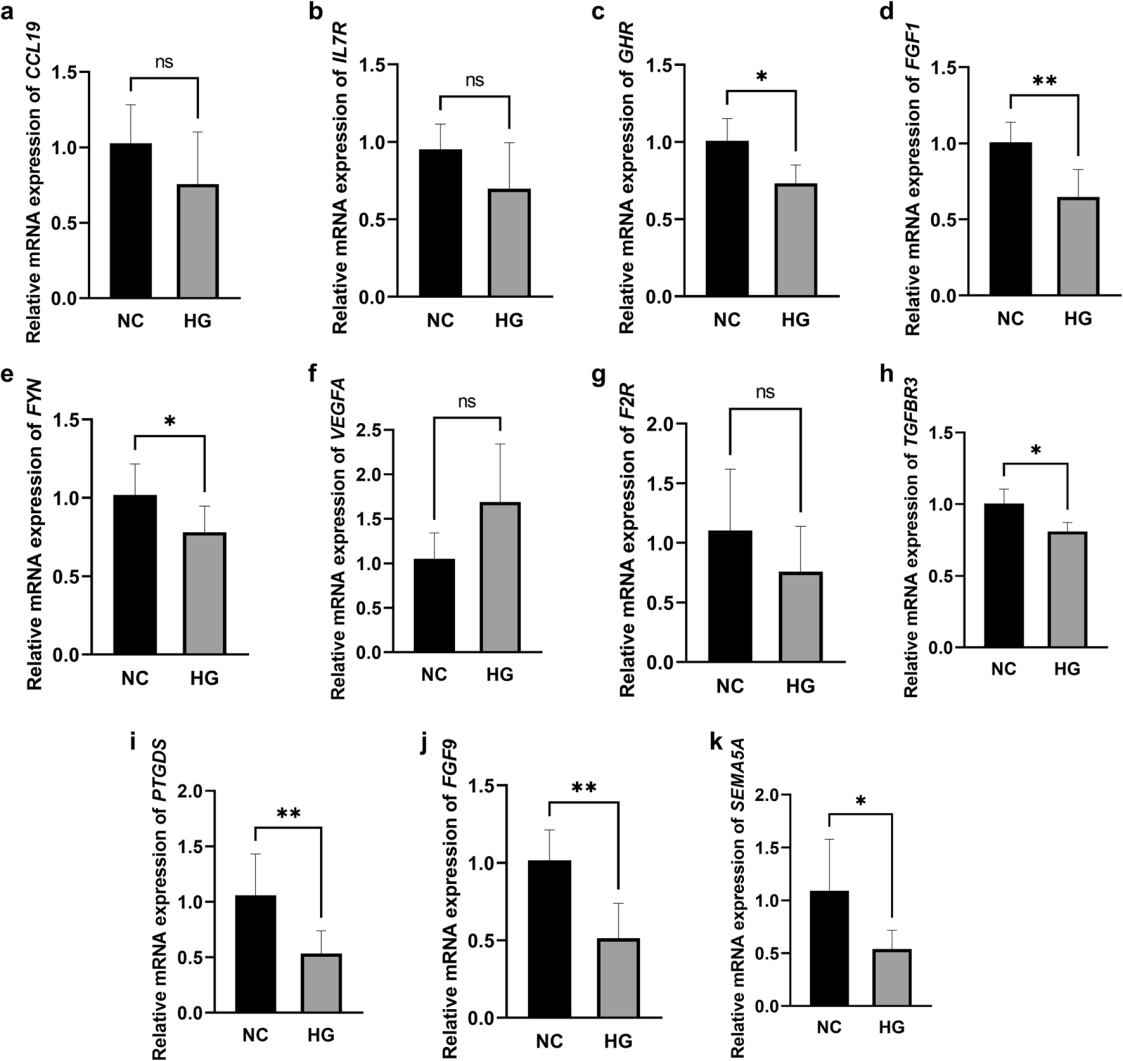
The relative mRNA expression levels of **a** *CCL 19*, **b** *IL7R*, **c** *GHR*, **d** *FGF1*, **e** *FYN*, **f** *VEGFA*, **g** *F2R*, **h** *TGFBR3*, **i** *PTGDS*, **j** *FGF9*, and **k** *SEMA5A* in the NC and HG group. NC, normal control; HG, high-glucose; ns: *P* > 0.05; *P < 0.05; **P < 0.01

Based on the clinical significance of DEIRGs, *CCL19*, *IL7R*, *GHR*, *FGF1*, *FYN*, *VEGFA*, *F2R*, *TGFBR3*, *PTGDS*, *FGF9*, and *SEMA5A* were selected to perform RT-qPCR to further validate their expression in podocytes. The results showed that the relative mRNA expression levels of *GHR*, *FGF1*, *FYN*, *TGFBR3*, *PTGDS*, *FGF9*, and *SEMA5A* were significantly downregulated in the HG group compared with the NC group (Fig. 4a–k). Therefore, *GHR*, *FGF1*, *FYN*, *TGFBR3*, *PTGDS*, *FGF9*, and *SEMA5A* were defined as experimentally validated DEIRGs.

### Construction of the predicted miRNAs-experimentally validated DEIRGs-TFs network

**Fig. 5:**
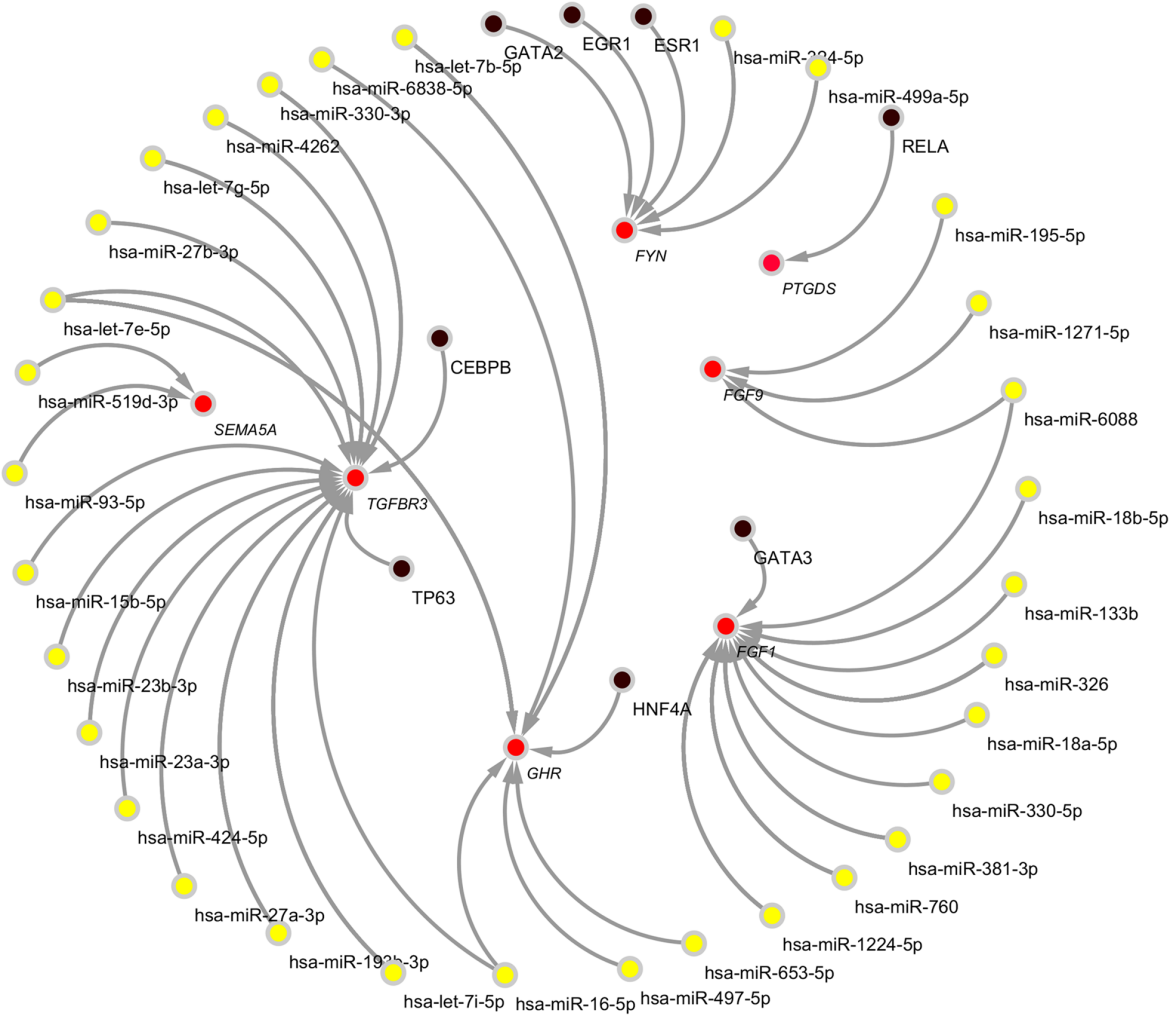
The predicted miRNAs-experimentally validated DEIRGs-TFs network. The red circles represent validated differentially expressed immune-related genes (DEIRGs). The yellow circles represent microRNAs (miRNAs). The black circles represent transcription factors (TFs)

The enriched miRNAs in TargetScan and miRDB databases were intersected and 110 common miRNAs were recorded. Similarly, the enriched TFs in ChEA and JASPAR databases were intersected and 8 common TFs were recorded. The network among miRNAs, target genes, and TFs can be seen in Fig. 5.

### Identification of the drugs target to experimentally validated DEIRGs

**Fig. 6:**
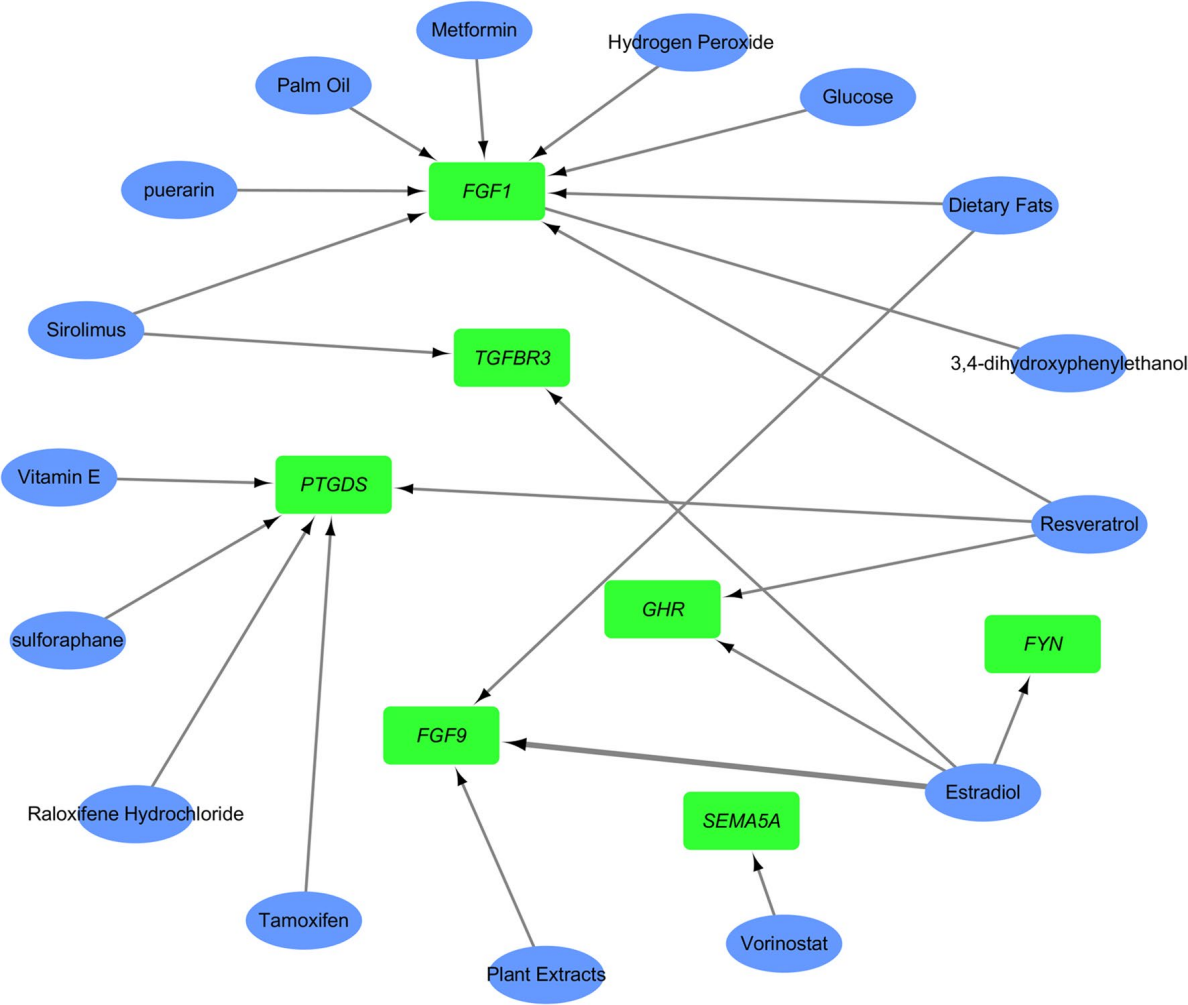
Drugs-DEIRGs interaction network. The green round rectangles represent downregulated differentially expressed immune-related genes (DEIRGs) in DKD. The blue ellipses represent targeted therapy drugs. The black arrows represent that the drugs could increase the expression of target genes

The CTD database was used to construct the network between drugs and the experimentally validated DEIRGs associated with DKD. The results showed that various drugs or chemicals such as estradiol, and resveratrol could regulate the abnormal mRNA expression levels of validated DEIRGs (Fig. 6).

## Discussion

Accumulating evidence from experimental and clinical studies has demonstrated that immune response is associated with the progression of DKD [18]. In the present study, a total of 12 DEIRGs associated with DKD were identified, including 3 upregulated DEIRGs (*CCL19*, *IL7R*, and *TRBC1*) and 9 downregulated DEIRGs (*GHR*, *FGF1*, *FYN*, *VEGFA*, *F2R*, *TGFBR3*, *PTGDS*, *FGF9*, and *SEMA5A*). The results in the Woroniecka Diabetes Glom dataset of Nephroseq database also proved that the expression levels of *CCL19* and *IL7R* were significantly increased, whereas the expression levels of *GHR*, *FGF1*, *FYN*, *VEGFA*, *F2R*, *TGFBR3*, *PTGDS*, *FGF9*, and *SEMA5A* were significantly decreased in the DKD group. In parallel, the up-regulation of *CCL19*, *IL7R* and the down-regulation of *GHR*, *FGF1*, *FYN*, *VEGFA*, *F2R*, *TGFBR3*, *PTGDS*, *FGF9*, and *SEMA5A* were correlated with the deterioration of renal function. Thus, *CCL19*, *IL7R*, *GHR*, *FGF1*, *FYN*, *VEGFA*, *F2R*, *TGFBR3*, *PTGDS*, *FGF9*, and *SEMA5A* were hub DEIRGs verified in clinical significance. To further confirm the results from bioinformatics analysis, we performed corresponding in vitro experiments.

And our experimental results confirmed that the relative mRNA expression levels of *GHR*, *FGF1*, *FYN*, *TGFBR3*, *PTGDS*, *FGF9*, and *SEMA5A* were significantly down-regulated in the HG group. Thus, these experimentally validated DEIRGs could be therapeutic targets for DKD. It has been well established that the activation of the immune inflammatory response can lead to podocyte injury and disrupt the integrity of the glomerular filtration barrier [19] and subsequently induce further deterioration of the renal function and glomerular sclerosis [20]. Consequently, changes in DEIRGs may be useful for timely recognition and treatment of DKD. Therefore, we also explored miRNAs, TFs, and drugs that may regulate the expression levels of DEIRGs.

It has been shown that podocytes express *GHR* when exposed to growth hormone (GH) [21]. Past research has revealed that under the condition of different chronic kidney diseases, including DKD, the expression level of renal *GHR* was decreased both in humans and in rodents. And the increase of *GHR* level was associated with the increase of GFR and the decrease of serum creatinine [22]. However, there are also a few converse opinions. A previous study indicated that chronic exposure to high doses of GH may induce glomerulosclerosis and increased proteinuria [23]. Another study also showed that increased renal expression of the *GHR* was associated with nephropathy in poorly controlled type 1 diabetes [21]. Therefore, GH/*GHR* axis involved in the pathogenesis of DKD and maintaining the normal expression level of *GHR* may be a therapeutic target for DKD.

*FGF1* is a well-known mitogen and insulin sensitizer. A previous study has observed a significant reduction in the renal expression of *FGF1* in patients with DKD and in mouse models. And *FGF1* treatment can prevent the development of DKD by inhibiting the activation of inflammatory signaling cascades such as nuclear factor-κB and c-Jun N-terminal kinases signaling [24]. Meanwhile, *FGF1* treatment can also increase insulin sensitization, maintain normoglycemia, and prevent diabetic complications [25]. Similarly, *FGF9* can induce ERK and AKT phosphorylation and cAMP-response element binding protein and nuclear factor erythroid-derived 2-like 2 (Nrf2) activation to play the antioxidative function [26] and cardioprotective effect [27]. Our results also proved that the expression levels of *FGF1* and *FGF9* were decreased in DKD status. Considering our results together with past findings, we can speculate that the down-regulation of *FGF1* and *FGF9* indicates a weakening antioxidant capacity in DKD status, making the kidney vulnerable to oxidative stress injury. Therefore, *FGF1* and *FGF9* could be potential therapeutic targets for DKD.

*FYN* is a proto-oncogene. Previous research has shown that the lipid lowering drug-fenofibrate can protect against DKD by activating Akt2/GSK-3β/*FYN*/Nrf2 antioxidants [28]. Another study also proved that sulforaphane can ameliorate DKD via GSK-3β/*FYN*/Nrf2 signaling pathway [29]. Therefore, *FYN* maybe a promising target in the treatment of DKD. Our results from gene-targeted drugs showed that sulforaphane can also target *PTGDS* to play a protective role in DKD. *PTGDS* was downregulated by DNA methylation [30]. Therefore, the downregulation of *PTGDS* in DKD represents an increased DNA methylation which is closely associated with aging and senescence [31]. Thus, improving the expression level of *PTGDS* by altering DNA methylation may not only contribute to the control of DKD but also help in delaying or alleviating the consequences of aging.

*TGFBR3* is a membrane proteoglycan that functions as a co-receptor with other transforming growth factor receptors. Soluble *TGFBR3* may inhibit transforming growth factor-β (TGF-β) signaling [32]. It has been extensively shown that TGF-β is highly activated in glomeruli, tubules, and tubulointerstitium in both patients and animal models with DKD [33]. Hyperglycemia and the formation of advanced glycation end products (AGEs), angiotensin II, and reactive oxidative species induced by high glucose are capable of activating TGF-β [33]. And the activation of TGF-β is closely associated with renal fibrosis and the deterioration of renal function [34]. Thus, the decrease of *TGFBR3* proved by the present study may lead to a decrease in its inhibition on TGF-β, which would further increase the expression level of TGF-β and aggravate renal impairment. Therefore, the current study supports the loss of *TGFBR3* as one mechanism for the development of DKD.

*SEMA5A* is a member of the semaphorin family which involved in neuronal and vascular development [35]. *SEMA5A* can also increase endothelial cell proliferation and decrease cell apoptosis [35]. However, the role of *SEMA5A* in the occurrence and development of DKD has not been fully studied. We proved that the expression level of *SEMA5A* was significantly downregulated in DKD glomerulus by bioinformatics analysis. And our subsequent experimental findings confirmed the results predicted by bioinformatics analysis. Considering the effects of *SEMA5A* in endothelial cell proliferation, migration, apoptosis, neuronal and vascular development, we can speculate that the reduced expression of *SEMA5A* may involve not only in the progression of diabetic microvascular complications such as DKD, but also in other diabetic complications such as diabetic neuropathy and cognitive dysfunction. Therefore, regulation of *SEMA5A* may help alleviate diabetic complications.miRNA is an important component of the gene regulatory network, which can regulate the expression of the target gene at the post-transcriptional level [36]. TFs are sequence-specific DNA binding proteins capable of regulating transcription [37]. We identified 110 miRNAs and 8 TFs as regulators of DEIRGs. We could speculate that these regulators participate in the pathological process of DKD and targeting these regulators may help discover potential protective miRNAs or TFs in the future.

Gene-targeted drugs may affect the expression of DEIRGs to retard disease progression. To predict the potential effective drugs for DKD, we applied the CTD database to explore therapeutic agents that might reverse the abnormal expression of DEIRGs in DKD. After exploring the interactions between validated DEIRGs and drugs associated with DKD, we found that 16 drugs could influence the expression of DEIRGs. Among these drugs, estradiol and resveratrol may occupy the most important positions because they can regulate four and three genes respectively. Estradiol is one of the most important sex steroid hormones secreted by the ovary [38]. Many studies have found that premenopausal women have a lower risk for developing DKD [39] because estradiol can attenuate glomerulosclerosis and tubulointerstitial fibrosis by reducing the synthesis of type I and type IV collagen [40–42]. Meanwhile, reduced concentrations of plasma estradiol exhibit a greater incidence and progression of DKD [39, 43]. And supplementation of 17β-estradiol markedly inhibited the progression of DKD [40, 44]. Our network pharmacology analysis showed that estradiol can improve the expression of abnormally down-regulated DEIRGs such as *GHR*, *TGFBR3*, *FYN*, and *FGF9*. Thus, estradiol can inhibit the occurrence or development of DKD by acting on at least 4 gene targets. Our findings also showed that resveratrol can prevent the development of DKD by reversing downregulated DEIRGs such as *FGF1*, *PTGDS*, and *GHR*. In essence, the chemical structure of resveratrol is similar to 17β-estradiol [45]. Consequently, resveratrol is structurally and functionally similar to estrogen [46]. Animal experiments have proved that resveratrol can prevent the progression of DKD by improving gut environment, reducing inflammatory response [47], improving kidney function, relieving proteinuria, ameliorating lipid metabolism, and inhibiting apoptosis [48]. Both resveratrol and 17β-estradiol can significantly reduce blood glucose, and protect pancreatic islet cell [46]. For the above reasons, we support the notion that proper 17β-estradiol intervention is an effective option in the management of DKD. It should also be noted that further experiments are needed to support whether DKD patients can benefit from DEIRGs intervention.

In conclusion, we identified 11 immune-related biomarkers for DKD, including 2 up-regulated DEIRGs (*CCL19*, *IL7R*) and 9 down-regulated DEIRGs (*GHR*, *FGF1*, *FYN*, *VEGFA*, *F2R*, *TGFBR3*, *PTGDS*, *FGF9*, *SEMA5A*) by bioinformatics analysis. The up-regulation of *CCL19*, *IL7R* and the down-regulation of *GHR*, *FGF1*, *FYN*, *VEGFA*, *F2R*, *TGFBR3*, *PTGDS*, *FGF9*, and *SEMA5A* were correlated with the deterioration of renal function. RT-qPCR showed that the relative mRNA expression levels of *GHR*, *FGF1*, *FYN*, *TGFBR3*, *PTGDS*, *FGF9*, and *SEMA5A* were significantly down-regulated in the high glucose-induced podocyte injury model. The enriched regulators for DEIRGs included 110 miRNAs and 8 TFs. Estradiol and resveratrol were the most enriched immune-related drugs in the treatment of DKD. The current findings may provide novel potential therapeutic targets for the precise diagnosis and immune-related therapies of DKD.

## Supplementary Information

**Additional file 1.** Differentially expressed genes in GSE1009 and GSE30528.

## Data Availability

The datasets analysed during the current study are available in the GEO and Nephroseq repository [Accession numbers: GSE1009 and GSE30528].

## Abbreviations

*CCL19*: C-C motif chemokine ligand 19
*IL7R*: Interleukin 7 receptor
*TRBC1*: T cell receptor beta constant 1
*GHR*: Growth hormone receptor
*FGF1*: Fibroblast growth factor 1
*FYN*: FYN proto-oncogene, Src family tyrosine kinase
*VEGFA*: Vascular endothelial growth factor A
*F2R*: Coagulation factor II thrombin receptor
*TGFBR3*: Transforming growth factor beta receptor 3
*PTGDS*: Prostaglandin-H2 D-isomerase
*FGF9*: Fibroblast growth factor 9
*SEMA5A*: Semaphorin 5A
