# Supplementary material for: Identification and validation of immune-related biomarkers and potential regulators and therapeutic targets for diabetic kidney disease
Source: BMC Med Genomics. 2023 May 1;16:90. doi: 10.1186/s12920-023-01519-6 (PMC10150481; doi:10.1186/s12920-023-01519-6)
Supplement: Supplementary file 1 — Additional file 1. Differentially expressed genes in GSE1009 and GSE30528. [file 12920_2023_1519_MOESM1_ESM.docx]

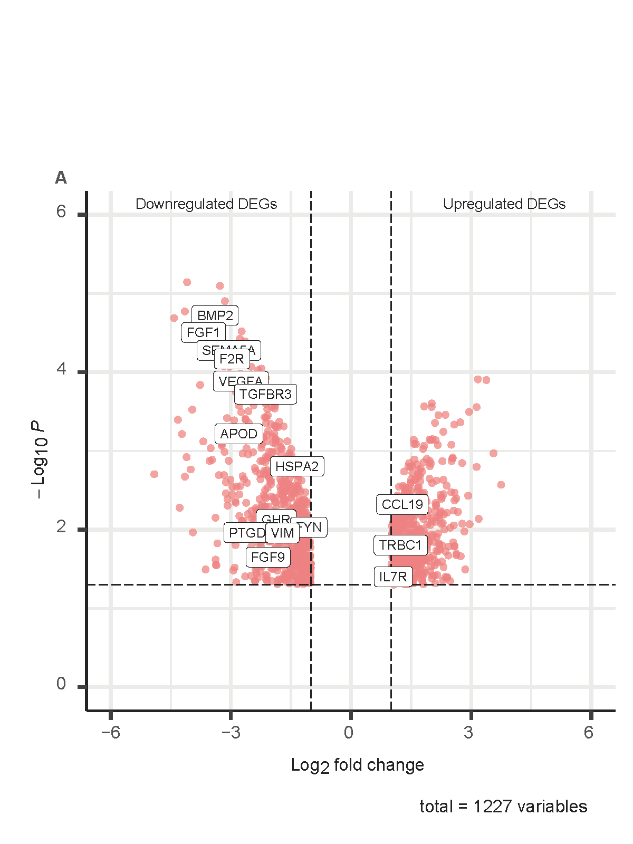

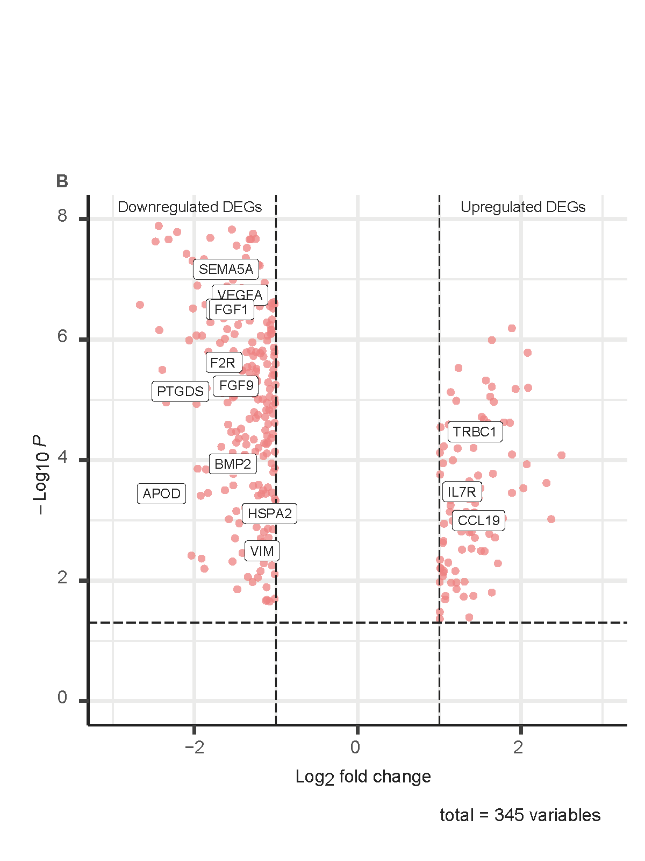


Supplementary Fig. 1 Differentially expressed genes (DEGs) in (A). GSE1009 and (B). GSE30528.

The red circles represent DEGs. The genes in black boxes represent differentially expressed immune-related genes (DEIRGs).

Supplementary Table 1. Interactions between genes and identified drugs

| Genes | Identified drugs | Co-expression |
| --- | --- | --- |
| FGF1 | 3,4-dihydroxyphenylethanol | 1 |
| FGF1 | Dietary Fats | 1 |
| FGF1 | Glucose | 1 |
| FGF1 | Hydrogen Peroxide | 1 |
| FGF1 | Metformin | 1 |
| FGF1 | Palm Oil | 1 |
| FGF1 | puerarin | 1 |
| FGF1 | Resveratrol | 1 |
| FGF1 | Sirolimus | 1 |
| FGF9 | Dietary Fats | 1 |
| FGF9 | Estradiol | 1 |
| FGF9 | Plant Extracts | 1 |
| FYN | Estradiol | 1 |
| GHR | Estradiol | 1 |
| GHR | Resveratrol | 1 |
| PTGDS | Raloxifene Hydrochloride | 1 |
| PTGDS | Resveratrol | 1 |
| PTGDS | sulforaphane | 1 |
| PTGDS | Tamoxifen | 1 |
| PTGDS | Vitamin E | 1 |
| SEMA5A | Vorinostat | 1 |
| TGFBR3 | Estradiol | 1 |
| TGFBR3 | Sirolimus | 1 |

Co-expression values of “1” represent that the drugs could increase the expression of target genes.
